# Comprehensive Phylogenetic Diversity of [FeFe]-Hydrogenase Genes in Termite Gut Microbiota

**DOI:** 10.1264/jsme2.ME13082

**Published:** 2013-11-15

**Authors:** Hao Zheng, Dylan Bodington, Chong Zhang, Kazuhiko Miyanaga, Yasunori Tanji, Yuichi Hongoh, Xin-Hui Xing

**Affiliations:** 1 Department of Chemical Engineering, Tsinghua University, 607 Yingshi Building, Beijing 100084, China; 2 Department of Biological Sciences, Graduate School of Biosciecnce and Biotechnology, Tokyo Institute of Technology, Tokyo 152–8550, Japan; 3 Department of Bioengineering, Graduate School of Biosciecnce and Biotechnology, Tokyo Institute of Technology, Yokohama 226–8501, Japan

**Keywords:** biomass energy, symbiosis, gut bacteria, hydrogenase, hydrogen

## Abstract

Phylogenetic diversity of [FeFe]-hydrogenase (HydA) in termite guts was assessed by pyrosequencing PCR amplicons obtained using newly designed primers. Of 8,066 reads, 776 *hydA* phylotypes, defined with 97% nucleotide sequence identity, were recovered from the gut homogenates of three termite species, *Hodotermopsis sjoestedti*, *Reticulitermes speratus*, and *Nasutitermes takasagoensis*. The phylotype coverage was 92–98%, and the majority shared only low identity with database sequences. It was estimated that 194–745 *hydA* phylotypes existed in the gut of each termite species. Our results demonstrate that *hydA* gene diversity in the termite gut microbiota is much higher than previously estimated.

Hydrogen is generated in abundance as an end product of lignocellulose fermentation by anaerobic microbes in termite guts (6, 14). These gut microbes are essential to termites; they play crucial roles not only in digestion but also in nitrogen recycling and fixation (9, 12, 25). In this termite gut ecosystem, hydrogen is an important mediator of the symbiosis among microbes, as in many other anaerobic environments (10, 15, 19, 21, 22). In phylogenetically lower termites, the hindgut microbiota comprises both prokaryotes and protists (13), the latter of which are considered to be the primary agents of hydrogen production (18, 20). In phylogenetically higher termites, symbiotic gut protists are generally absent, and hydrogen production is solely attributable to gut bacteria, such as spirochetes (24, 27). Hydrogen production is achieved mainly by the action of hydrogenase, which reversibly catalyzes the formation of H_2_ from protons and electrons. Among several types of hydrogenase, [FeFe]-hydrogenase (HydA) has been found in both prokaryotes and eukaryotes, and in most cases is involved in H_2_ production (16, 18, 23, 28). [FeFe]-hydrogenase can be functionally expressed in heterologous cells of bacteria to analyze its properties and enhance H_2_ production (18, 28). In this study we aimed to measure the phylogenetic diversity of *hydA* genes in the gut microbiota of termites in an effort to generate a good resource of novel hydrogenase genes useful for more efficient hydrogen production.

DNA was extracted from the entire guts of the lower termites *Hodotermopsis sjoestedti* (abbreviated as Hs in this study) (family Termopsidae), *Reticulitermes speratus* (Rs) (family Rhinotermitidae), and the wood-feeding higher termite *Nasutitermes takasagoensis* (Nt) (family Termitidae), as described previously (26). We designed a new primer set, targeting *hydA* families, 3, 6, 7, and “FDH-linked”, all of which have previously been identified and designated in a metagenomic study of termite gut microbiota (27). Based on the alignment of *hydA* sequences in public databases, primers Fe-P1f (5′-TTYACHTCCTGYTGYCCNGSHTGG-3′) and Fe-P3r (5′-CADCCDCCNGGRCANGCCAT-3′) were designed in the conserved H-cluster segments P1 and P3, respectively, with degeneracy decreased according to the base frequency. This primer set flanks the region containing the conserved H-cluster segment P2, which is useful as an identifier of *hydA* to discriminate it from other oxidoreductases.

PCR amplification of *hydA* from the termite gut samples was successful with this new primer set. PCR products of the expected size (0.62 kb) were purified and cloned, and restriction fragment length polymorphism (RFLP) analysis was performed. Fifty-five randomly chosen *hydA* clones from each termite sample were analyzed, and 25, 27, and 23 RFLP patterns were found in samples Hs, Rs, and Nt, respectively. A representative clone or clones for each RFLP pattern were sequenced using an ABI 3730 genetic analyzer. When more than one representative clone of an RFLP pattern was sequenced, all representative clone sequences were identical. The detailed methods are described in the supplemental materials.

After removal of non-*hydA* sequences, 73 unique sequences remained. These remaining sequences were sorted into operational taxonomic units (OTUs) with a criterion of 97% nucleotide sequence similarity, using the furthest-neighbor joining method as shown in Table 1. This criterion was used throughout this study unless otherwise stated. The sequences have been deposited in GenBank/DDBJ/EMBL under accession numbers JF802513–JF802574.

Since Good’s coverage of OTUs obtained by the Sanger sequencing analysis was as low as 65.5–70.9% (Table 1), we additionally performed pyrosequencing to comprehend the diversity of *hydA*, using a 454 Life Sciences Genome Sequencer FLX Titanium (Roche, Penzberg, Germany). The raw sequence reads and quality files have been deposited in the NCBI sequencing read archive under project number SRA047832. The reads were analyzed using a program package, Quantitative Insights into Microbial Ecology (QIIME) (7).

The 454 reads with low quality and/or shorter than 250 bases were discarded. Indels, which were most probably sequencing errors, were then corrected using the FunGene module on the Ribosomal Database Project web site (http://rdp.cme.msu.edu/) by comparing them with known HydA amino acid sequences. Sequences that shared <40% similarity with known HydA amino acid sequences were excluded from the downstream analyses. The remaining 8,066 sequences were sorted into 776 OTUs. From Hs, Rs, and Nt samples, 213, 148, and 421 OTUs were identified respectively. Both Shannon and Simpson indices were highest in the Nt sample (Table 1) and the rarefaction curves also indicated the highest diversity in Nt (Fig. 1). The numbers of OTUs based on translated amino acid sequences are also indicated in Table 1.

The coverage was as high as 92.1–98.2% in these samples (Table 1), but the rarefaction curves suggest that further sampling is required to obtain all OTUs present in the samples (Fig. 1). Therefore, we estimated the entire OTU richness using the Chao1 method (8), and the expected OTU numbers were 373, 194, and 745 for Hs, Rs, and Nt, respectively. The estimated Chao1 value for Nt should be regarded as the minimum, because the Chao1 estimator is sensitive to the sampling effort and the value has not yet reached the plateau for Nt (data not shown) (17). The higher phylogenetic diversity of *hydA* in a higher termite agreed with previous reports (3, 4).

The number of observed OTUs, coverage, Shannon index, and Chao1 richness were considerably higher than both those calculated in our clone analysis and those found in previous studies by Ballor and Leadbetter (3, 4). In their study of gut homogenate samples from three lower and six higher termite species, the number of observed OTUs (defined with 97% amino acid sequence similarity) was 16–44, Chao1 richness 17–68, and Shannon index 2.12–3.53. The difference is mostly attributable to the higher sequencing effort, and partly attributable to the wider target of the primer set used here. Even when 70% sequence similarity was adopted for the criterion of OTU, 193 *hydA* OTUs were identified from the three termite species (Table 1).

Fig. 2 shows an outline of the phylogenetic diversity of *hydA* obtained in the present study. The full tree is presented in Fig. S1. Several sequence clusters belonged to families 3 and 7, while others might form novel families. The division of the families was possibly caused by the low resolution of the tree or the expanded diversity found in this study. Since the majority of termite gut microbiota are as yet unculturable, only a few bacterial species in termite guts have been identified to possess *hydA: Treponema azotonutricum*, *Treponema primitia* (2), *Spirochaeta coccoides* (1), and *Candidatus* Endomicrobium trichonymphae phylotype Rs-D17 (11). In the present study, several OTUs formed clusters with reference database sequences (Fig. S1); those OTUs might have derived from bacteria belonging or related to the genera *Treponema*, *Spirochaeta*, *Clostridium*, *Eubacterium*, *Acetivibrio*, *Syntrophomonas*, *Parabacteroides*, *Odoribacter*, and *Desulfovibrio*, all of which have been found in termite gut microbiota based on 16S rRNA clone analyses (13). Several OTUs from the lower termite *H. sjoestedti* showed high similarity to those of gut protists. Other OTUs shared only low identity with the reference sequences, including those from termite guts (Fig. S1) (3, 4).

Phylogenetic relationships of *hydA* among different termite hosts are shown in Fig. 3 and S1. The OTUs from the same host termite species tend to cluster together. In addition, those from congeneric termites, *e.g.*, *Nasutitermes takasagoensis* and *N. ephratae*, are closely related. These are in concordance with previous reports that 16S rRNA phylotypes from the same termite species or genus tend to cluster together (13). The congruence of *hydA* sequence similarity and termite host phylogeny has also been shown in a previous study (3).

In this study, it was revealed that the gut microbiota of a single termite species potentially contains 194–745 *hydA* phylotypes. Recently, Boucias *et al.* (5) estimated, by 454 pyrosequencing analysis of 16S rRNA genes, that the lower termite *Reticulitermes flavipes* harbors nearly 5,000 bacterial species. Therefore, it may not be surprising that several hundred phylotypes of *hydA* were found from a single termite species. It is noteworthy that there were only a few overlaps of *hydA* phylotypes among the host termite species. This implies an enormous diversity of *hydA* in the total termite gut microbiota on Earth, considering that there are nearly 300 genera and 3,000 species of termites (Constantino, R.; http://164.41.140.9/catal/). These *hydA* genes might be useful in designing a recombinant hydrogenase to produce hydrogen with better efficiency.

## Supplementary Information



## Figures and Tables

**Fig. 1:**
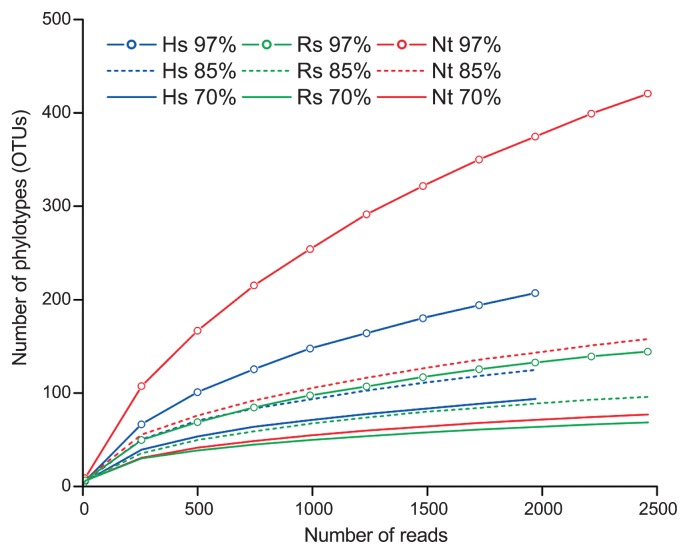
Rarefaction curves of *hydA* OTUs obtained from the gut of three termite species. Hs: *Hodotermopsis sjoestedti*, Rs: *Reticulitermes speratus*, Nt: *Nasutitermes takasagoensis*.

**Fig. 2:**
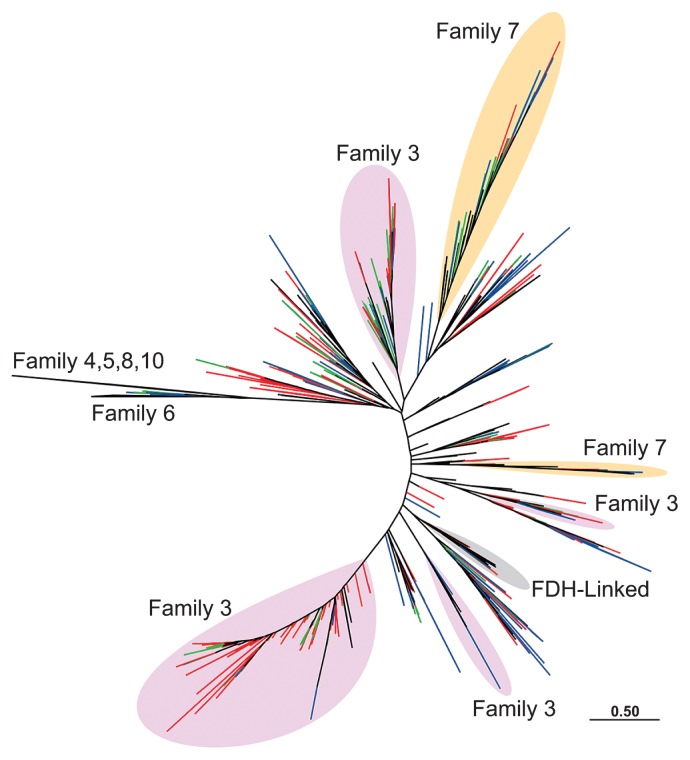
A maximum-likelihood tree based on deduced amino acid sequences showing the phylogenetic diversity of *hydA* obtained in this study. The tree was constructed using 102 amino acid sites. The branches were marked with different colors corresponding to their respective host termites: *Hodotermopsis sjoestedti* (blue), *Reticulitermes speratus* (green), *Nasutitermes takasagoensis* (red). The *hydA* families designated in previous studies (2, 27) are indicated in different colors. The method for the tree construction is described in the supplemental materials. The full tree is shown in Fig. S1.

**Fig. 3:**
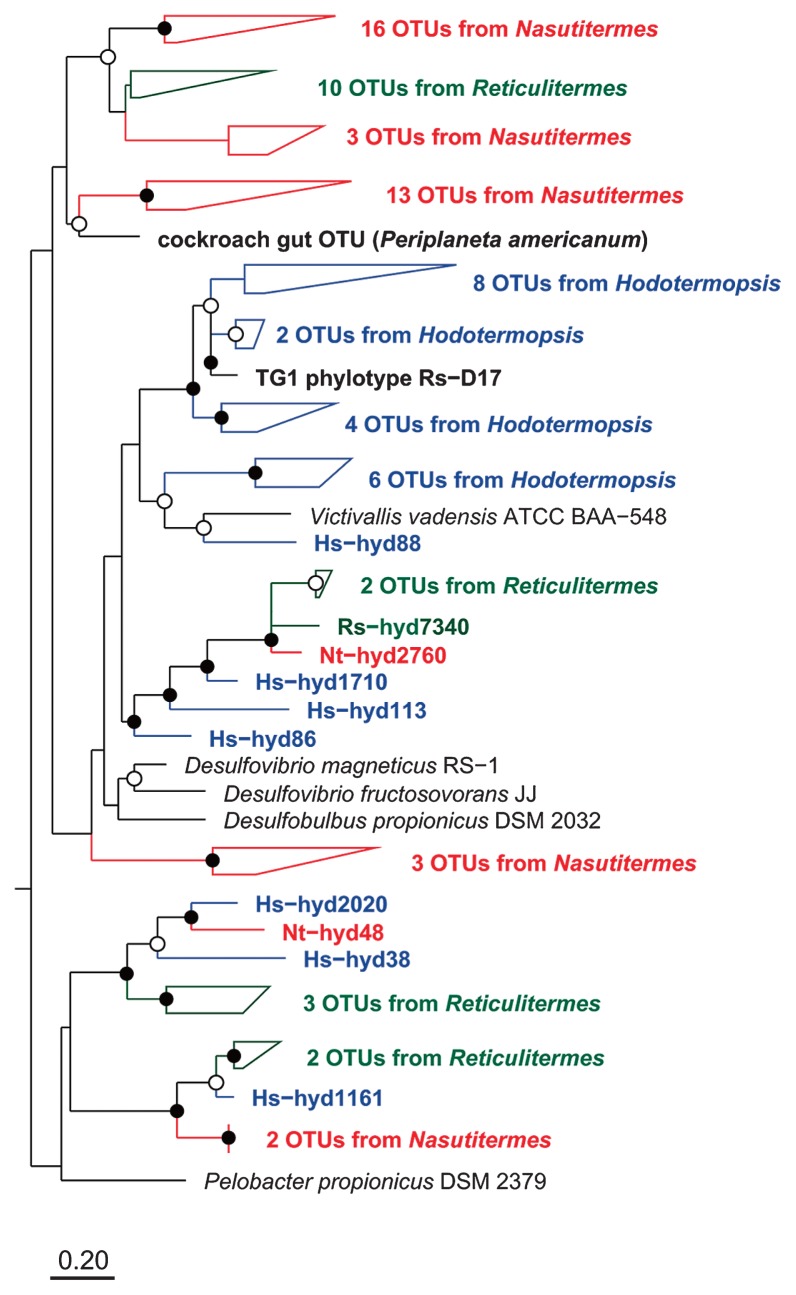
A representative part of a maximum-likelihood tree of *hydA* based on deduced amino acid sequences. Open and closed circles at the nodes indicate bootstrap confidence values of 50–74% and 75–100%, respectively. The full tree is shown in Fig. S1.

**Table 1:** Diversity of *hydA* nucleotide sequences in termite gut microbiota

	Sanger sequencing			454 pyrosequencing^c^				
		
	Hs	Rs	Nt	Hs	Rs	Nt	Total	Shared OTUs
Number of analyzed sequences	55	55	55	2388	2991	2687	8066	
Mean sequence length (base)	681	657	648	349	341	340	343	
OTUs (97% similarity)	20	21	20	213 (276)	148 (225)	421 (419)	776	6
OTUs (85% similarity)	16	20	11	128	98	158	373	11
OTUs (70% similarity)	14	15	6	96	70	77	193	50
Chao1	106	41	97	373 (511)	194 (333)	745 (636)		
Shannon	2.98	2.99	2.93	4.18 (4.75)	4.23 (4.50)	6.58 (5.30)		
Simpson^a^	0.99	0.98	0.98	0.74	0.86	0.97		
Coverage^b^ (%)	65.5	70.9	67.3	95.6	98.2	92.1		

a Simpson’s index of diversity, indicated as 1-D.

b Good’s coverage estimator: (1-n/N) × 100, where n is the number of phylotypes represented by only one sequence and N is the total number of sequences.

c The values in parentheses are those calculated from amino acid sequences.
