# Supplementary material for: Comprehensive Phylogenetic Diversity of [FeFe]-Hydrogenase Genes in Termite Gut Microbiota
Source: Microbes Environ. 2013 Nov 15;28(4):491–4. doi: 10.1264/jsme2.ME13082 (PMC4070709; doi:10.1264/jsme2.ME13082)
Supplement: Supplementary file 1 [file 28_491_s1.pdf]

Supplemental Materials

Materials and Methods

Termites and DNA extraction

The entire guts were removed from 5–20 randomly chosen workers per colony by using sterilized forceps. DNA was extracted from the gut homogenates using the Isoplant II kit (Nippon Gene, Tokyo Japan), which chemically lyses bacterial cell walls and membranes with benzyl chloride. The extracts were further purified using the DNeasy tissue kit (Qiagen) as described previously (6).

PCR, cloning, and RFLP analysis

The *hydA* gene was amplified from the extracted DNA by PCR with EX-*Taq* polymerase (Takara, Shiga, Japan). The reaction conditions were 35 cycles of 94°C for 30 s, 58°C for 30 s, and 72°C for 65 s. PCR products of the expected size (approximately 0.62 kb) were separated by agarose gel electrophoresis and purified using the Wizard SV Gel and Clean-Up System (Promega, Madison, WI, USA). The purified PCR products were inserted into plasmids (pGEM-T Easy Vector, Promega) and cloned into *Escherichia coli* XL-1 Blue according to the manufacturer's instructions. The inserts were amplified by PCR using primers Fe-P1f and Fe-Pr3, and the products were digested with restriction enzymes *Msp* I and *Rsa* I (Nippon Gene) for restriction fragment length polymorphism analysis (RFLP). Digested fragments were visualized by electrophoresis in 2% agarose gels and were sorted according to their RFLP patterns.

Sanger sequencing

Plasmids were prepared from randomly chosen colonies and used as templates for DNA sequencing in an ABI3730 genetic analyzer. The obtained *hydA* nucleotide sequences were

subjected to Basic Local Alignment Search Tool X (BLASTx) analysis. The nucleotide sequences were translated to amino acid *in silico* and aligned by program MUSCLE (4) and corrected by manual inspection in ARB (5).

454 pyrosequencing

The *hydA* genes were amplified using the primer set Fe-P1f and Fe-P3r (see main text). The forward primer contained the 454 Life Sciences primer B sequences; the reverse primer the 454 Life Sciences primer A sequences. These are composed of unique 10 nucleotide sequences as barcodes to tag each PCR product. PCR reactions were carried out in triplicate 20-μl reactions with 0.4 μM forward and reverse primers, 1-μl template DNA, 250 nM dNTP and 1 × FastPfu Buffer. Thermal cycling consisted of initial denaturation at 95°C for 2 min followed by 25 cycles of denaturation at 95°C for 30 s, annealing at 55°C for 30 s, and extension at 72°C for 30 s, with a final extension of 5 min at 72°C. Replicate amplicons were pooled and purified using the AxyPrep™ DNA Gel Extraction Kit (Axygen, Union City, CA, USA) according to the manufacturer's instructions.

Amplicon DNA concentrations were measured using the Quant-iT PicoGreen dsDNA Kit (Life Technologies, Carlsbad, CA, USA)). After the measurement, the purified amplicons were combined in equimolar ratios into a single tube. Pyrosequencing was carried out in a 454 Life Sciences Genome Sequencer FLX Titanium instrument (Roche, Penzberg, Germany) by Shanghai Majorbio Bio-pharm Biotechnology Co.,Ltd. (Shanghai, China).

Bioinformatics

The raw barcoded pyrosequence reads were uploaded into a program package, Quantitative Insights into Microbial Ecology (QIIME) and processed (3). The sequences were screened using the following parameters: minimum quality score of 25, minimum sequence length of 250 bp, and no ambiguous bases in the entire sequence or mismatches in the primer sequence. The remainder were sorted by barcode into their respective samples. After trimming of the barcode and the primer

sequences, the sequences were imported into the FrameBot tool of the RDP FunGene analysis pipeline, which detects and corrects the frameshift errors caused by insertions and deletions when translating DNA sequences to protein sequences. Sequences were translated and aligned using the reference *hydA* set with a length cutoff of 80 amino acids and a percent identity cutoff of 40% to the reference set. Sequences which failed the translation were removed from downstream analyses.

Using QIIME, the nucleotide sequences were grouped into operational taxonomic units (OTUs) with 97% identity threshold. The sorted sequences were clustered by program UCLUST (http://www.drive5.com/), and a representative sequence from each OTU was selected. The taxonomy of the representatives was approximately assigned by NCBI Blastall (BLASTx) using the non-redundant protein sequences (nr) database with default settings.

Phylogenetic analysis

A maximum-likelihood tree was constructed using RAxML 7.0.3 implemented in ARB software (5), with a PROTMIX + WAG amino acid substitution model. An ambiguously aligned 102 amino acid residues were used. To evaluate the robustness of the topology, 100 bootstrap resamplings were performed. After that, HydA sequences from previous studies (1, 2) were added to the tree using the “Add species” function of ARB, based on a maximum parsimony criterion (5).

References

1. Ballor, N.R., and J.R. Leadbetter. 2012. Analysis of extensive [FeFe] hydrogenase gene diversity within the gut microbiota of insects representing five families of Dictyoptera. Microb. Ecol. 63: 585-595.

2. Ballor, N.R., and J.R. Leadbetter. 2012. Patterns of [FeFe] hydrogenase diversity in the gut microbial communities of lignocellulose-feeding higher termites. Appl. Environ. Microbiol. 78: 5368-5374.

3. Caporaso, J.G., J. Kuczynski, J. Stombaugh *et al.* 2010. QIIME allows analysis of high-throughput community sequencing data. Nat. Methods 7: 335-336.

4. Edgar, R.C. 2004. MUSCLE: multiple sequence alignment with high accuracy and high throughput. Nucl. Acids Res. 32: 1792-1797.

5. Ludwig, W., O. Strunk, R. Westram *et al.* 2004. ARB: a software environment for sequence data. Nucl. Acids Res. 32: 1363-1371.

6. Thongaram, T., S. Kosono, M. Ohkuma, Y. Hongoh, M. Kitada, T. Yoshinaka, S. Trakulnaleamsai, N. Noparatnaraporn, and T. Kudo. 2003. Gut of higher termites as a niche for alkaliphiles as shown by culture-based and culture-independent studies. Microbes Environ. 18: 152-159.

Legends to Supplemental Figures

Fig. S1: Phylogenetic relationships of *hydA* based on deduced amino acid sequences. The tree was constructed using a maximum likelihood method with 100 replicates of bootstrapping in RAxML. Sequences from the samples *H. sjoestedti* (Hs), *R. speratus* (Rs), *N. takasagoensis* (Nt) are presented in blue, green, and red, respectively. Sequences obtained by using Sanger sequencing start with "c". Open circles mark groupings supported by >50% bootstrap confidence and filled circles mark groupings supported by >75% bootstrap confidence. The shared OTUs is shown by attaching asterisk colored as above.

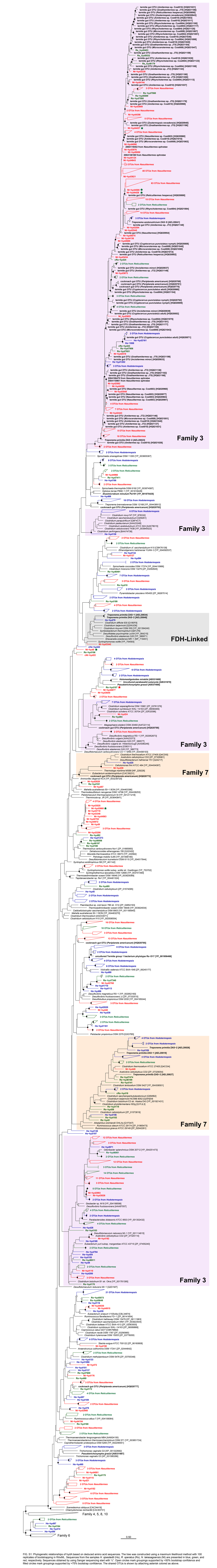

FIG. S1: Phylogenetic relationships of hydA based on deduced amino acid sequences. The tree was constructed using a maximum likelihood method with 100 replicates of bootstrapping in RAxML. Sequences from the samples *H. sjoestedti* (Hs), *R. speratus* (Rs), *N. takasagoensis* (Nt) are presented in blue, green, and red, respectively. Sequences obtained by using Sanger sequencing start with “c”. Open circles mark groupings supported by >50% bootstrap confidence and filled circles mark groupings supported by >75% bootstrap confidence. The shared OTUs is shown by attaching asterisk colored as above.
